# Latent class analysis of multimorbidity patterns and associated outcomes in Spanish older adults: a prospective cohort study

**DOI:** 10.1186/s12877-017-0586-1

**Published:** 2017-08-18

**Authors:** Beatriz Olaya, Maria Victoria Moneta, Francisco Félix Caballero, Stefanos Tyrovolas, Ivet Bayes, José Luis Ayuso-Mateos, Josep Maria Haro

**Affiliations:** 1 Research, Innovation and Teaching Unit, Institut de Recerca Sant Joan de Déu, Carrer Dr. Antoni Pujadas, 42, Esplugues de Llobregat, 08830 Barcelona, Spain; 2 Parc Sanitari Sant Joan de Déu, Universitat de Barcelona, Fundació Sant Joan de Déu, Sant Boi de Llobregat, Barcelona, Spain; 3 Instituto de Salud Carlos III, Centro de Investigación Biomédica en Red de Salud Mental (CIBERSAM), Madrid, Spain; 4 Department of Psychiatry, Universidad Autónoma de Madrid, Madrid, Spain; 5 Department of Psychiatry, Instituto de Investigación Sanitaria Princesa (IP), Hospital Universitario de La Princesa, Madrid, Spain

**Keywords:** Clusters, Multimorbidity, Follow-up, Older adults, Cognition, Quality of life, Disability, Use of health services

## Abstract

**Background:**

This study sought to identify multimorbidity patterns and determine the association between these latent classes with several outcomes, including health, functioning, disability, quality of life and use of services, at baseline and after 3 years of follow-up.

**Methods:**

We analyzed data from a representative Spanish cohort of 3541 non-institutionalized people aged 50 years old and over. Measures were taken at baseline and after 3 years of follow-up. Latent Class Analysis (LCA) was conducted using eleven common chronic conditions. Generalized linear models were conducted to determine the adjusted association of multimorbidity latent classes with several outcomes.

**Results:**

63.8% of participants were assigned to the “healthy” class, with minimum disease, 30% were classified under the “metabolic/stroke” class and 6% were assigned to the “cardiorespiratory/mental/arthritis” class. Significant cross-sectional associations were found between membership of both multimorbidity classes and poorer memory, quality of life, greater burden and more use of services. After 3 years of follow-up, the “metabolic/stroke” class was a significant predictor of lower levels of verbal fluency while the two multimorbidity classes predicted poor quality of life, problems in independent living, higher risk of hospitalization and greater use of health services.

**Conclusions:**

Common chronic conditions in older people cluster together in broad categories. These broad clusters are qualitatively distinct and are important predictors of several health and functioning outcomes. Future studies are needed to understand underlying mechanisms and common risk factors for patterns of multimorbidity and to propose more effective treatments.

**Electronic supplementary material:**

The online version of this article (doi:10.1186/s12877-017-0586-1) contains supplementary material, which is available to authorized users.

## Background

Chronic conditions and non-communicable diseases are the leading cause of morbidity and disability worldwide [1]. The co-occurrence of two or more conditions, or multimorbidity, is especially common in elderly people. Approximately two out of three persons at retirement age suffer from at least two chronic diseases [2]. The therapeutic management of multimorbidity is often complex because people suffering from multimorbidity present high treatment burden, poorer health outcomes, problems in independent living [3, 4] and higher rates of mortality [5].

The classical single-disease paradigm might not be adequate for patients with multiple chronic conditions [6]. Studies have focused on different approaches to assess multimorbidity. Counting the number of diseases has been a commonly used method [7, 8] but results could be affected by the number of chronic conditions considered. One variant is the conditional count [9, 10]. For example, people with arthritis have, on average, 3.8 co-morbid conditions, compared with 1.8 conditions among persons without arthritis [9]. Other approaches include the most prevalent pair combinations. Diabetes and cataract, or asthma and chronic obstructive pulmonary disease (COPD), are especially prevalent and associated with poor levels of quality of life and high disability [4]. However, these approaches might not adequately capture larger clusters of conditions linked to greater burden. In recent years, the co-occurrence of chronic conditions has been addressed with cluster analysis methods as they can help to identify broad comorbidity patterns.

Islam et al. [6] compared distinct methods to assess multimorbidity patterns in a community sample of elderly people, including the most frequent pairs and triplets of comorbid diseases, cluster analysis, principal component analysis and latent class analysis (LCA). They found considerable consistency across the analytic methods although some differences arose, mainly due to the underlying methodology. Cluster analysis is based on distance measures, whereas principal components and LCA are based on correlations, with similarities between the last two approaches [6].

Few studies have analyzed multimorbidity patterns using LCA. LCA is based on structural equation modeling which allows identification of latent groups based on a set of observed variables. It can be useful in describing how people are clustered according to patterns of chronic conditions and identifying the main differences between these groups in terms of sociodemographics, functioning and clinical characteristics. Whitson et al. [11] reported six latent groups in a community dwelling sample of people aged 65+. These multimorbidity groups were significantly associated with emergency department use and hospitalization over 1 year. However, the authors reported also high misclassification errors as one limitation of the study. There is still a need to understand how chronic conditions are clustered together, whether these multimorbidity patterns are reliable and valid, and determine the impact on health and functioning outcomes at baseline and longitudinally. Moreover, there is also a need for general-population studies to ensure the external validity of multimorbidity patterns [12].

The aim of this study was to investigate whether eleven common chronic conditions cluster together in a Spanish representative sample of people aged 50 and older according to their pattern of co-occurrence. LCA was conducted to identify latent groups of people based on a set of observed variables [13]. These latent groups were then described in terms of sociodemographic characteristics, co-morbid conditions, quality of life, disability, functioning and cognitive function. The second part of the analysis involved the study of the effect of these latent co-morbid groups on several outcomes measured at baseline and at three-year follow-up.

## Methods

### Design and setting

The present study used data from “*Edad con Salud*”, a longitudinal, nationally representative survey of adult, non-institutionalized people in Spain. The first wave was part of the Collaborative Research on Ageing in Europe (COURAGE in Europe) study [14]. A stratified, multistage clustered design was used and people over 50 and 80 years old were oversampled. Strata included all Autonomous Communities in Spain (except Ceuta and Melilla). Data on households were provided by the Spanish Statistical Office. A total of 4753 persons participated in face-to-face structured interviews conducted at their homes between 2011 and 2012. The final response rate at baseline was 69.9%. The second wave took place after approximately 3 years (2014–2015). 2528 participants (53.2%) completed the interview at follow-up, 259 (5.5%) had died, 862 (18.1%) declined to participate in the second assessment, and 23.2% did not participate for other reasons (e.g., unable to locate, institutionalization).

The present analysis focused on people aged 50 years of age and older who completed a non-proxy interview at baseline (*n =* 3625). Of these, 1970 participated in an interview in the second wave. In a small proportion (*n =* 84), a proxy responded to the second assessment because the original participant presented some evidence of cognitive deterioration. Since proxy interviews were much shorter, we excluded these proxy interviews at follow-up, resulting in a final *n* of 3541 participants at baseline, of whom 1886 also participated in the second assessment.

### Measures

Presence or absence of eleven chronic conditions at baseline was considered. Participants reported whether they had received a medical diagnosis during the previous 12-months of depression, arthritis, asthma, COPD, angina, stroke, hypertension, diabetes, edentulism and cataract. Other symptom questions were added to the interview based on the WHO-SAGE protocol [15] and the adapted version of the World Health Organization Composite International Diagnostic Interview (CIDI) for depression [16]. These additional questions allowed us detect undiagnosed cases. Algorithms were implemented [17, 18] and an individual was considered to have one of these conditions if he/she met criteria for at least the self-reported diagnosis, or the diagnosis made according to symptoms. The presence of hypertension was based on self-reported diagnosis or presence of systolic blood pressure ≥ 140 mmHg or diastolic blood pressure ≥ 90 mmHg [19, 20] measured at the time of the interview. Blood pressure was measured twice with less than a 1 minute interval using an arm blood pressure monitor and taking the average of the measurements. Interviewers recorded participants’ height and weight using a stadiometer and a routinely calibrated electronic weighing scale, respectively. Body Mass Index (BMI) was calculated as weight (in kilograms) divided by the square of height (in meters). A BMI of 30 or higher was used as cut-off point for obesity [21].

Self-reported demographic variables at baseline included age, gender, years of schooling, quintiles of household income (with the first quintile indicating lowest level and the fifth the highest), and marital status (never married, married or currently cohabiting, separated or divorced, and widowed).

Respondents were asked to recall a list of words three times immediately and once after a short delay which was filled with other cognitive tests (Consortium to Establish a Registry for Alzheimer’s Disease) [22]. The psychometric properties of the Word List Learning task have previously been established [23, 24]. A composite score was calculated as the sum of the number of correct words, ranging from 0 to 40, with higher scores indicating better memory. Participants were asked to name as many animals as possible in 1 minute, both at baseline and follow-up. Animal naming tasks are considered a measure of verbal fluency whereas word-list recall is regarded as a measure of verbal memory.

Disability was assessed with the 12-item version of the World Health Organization Disability Assessment Schedule 2.0 (WHODAS 2.0) [25] which evaluates functioning in six life domains. A total score was obtained as the sum of the items and transformed into a 0 to 100 scale (higher scores indicating greater disability). Presence or absence of difficulties in independent living was evaluated through activities of daily living (ADLs) and instrumental activities of daily living (IADLs). ADLs describe a set of daily self-care activities and assess the need for help with personal care activities such as eating, bathing and dressing; IADLs describe higher-level functioning considered necessary to live independently (using transportation, housekeeping, or preparing food). ADLs and IADLs difficulties were present if the person answered *severe* or *extreme/cannot do it* to any of the questions. Quality of life was measured at baseline and follow-up with the WHOQOL-AGE [26], a modified version of the World Health Organization Quality of Life instrument (WHOQOL) adapted for the elderly population. It has 13 items and a global score can be obtained, ranging from 0 (minimum quality of life) to 100 (maximum quality of life). The total number of visits to any health professional in the last year as well as whether the participant had been hospitalized in the previous 12 months (yes/no) was also recorded at baseline and at follow-up.

### Statistical analysis

Latent Class Analysis (LCA) was conducted on the 3541 participants at baseline using the Stata plugin [27]. Eleven chronic health conditions (arthritis, asthma, COPD, angina, stroke, hypertension, diabetes, edentulism, cataract, depression, and obesity) were used as observed indicators. The optimal number of latent classes was determined using the adjusted Bayesian Information Criterion (BIC) [28] and the consistent Akaike Information Criterion (CAIC) [29], which have been shown to be more robust indicators of class enumeration with categorical outcomes [30]. The adjusted BIC and CAIC were used to compare several plausible models where the lowest values indicate the best fitting model.

Furthermore, interpretability and clinical judgment were used. After selecting the best model, each participant was assigned to one class according to his or her highest computed probability of membership. Average posterior probabilities above 70% indicate optimal fit [31]. Each latent class was labeled according to those chronic conditions whose prevalence exceeded the prevalence in the full cohort [11]. The final latent groups were compared in terms of co-morbidities and functioning and sociodemographic variables at baseline using chi-squared and Kruskal-Wallis tests for significance.

Missing data at baseline and follow-up was handled using a Stata command for Imputation by Chained Equations (ICE) [32] assuming missing-at-random (MAR). The imputation model included all the variables used in the regression models plus other auxiliary variables (variables not included in the analysis model but which are potential predictors of missingness [33]) (see Additional file 1: Table S1). Since the imputed values of the continuous variables lay outside the observed data range, they were transformed to normality before imputing their values and then converted back to the original variables following multiple imputation [34]. LCA was performed without imputing missing values in the eleven indicators because LCA is not supported by *mim* command [35] for analyzing multiply imputed datasets. Instead, missing data in one of the indicators was tolerated and handled with a full-information maximum likelihood (FIML) technique, assuming MAR [27]. Additional file 1: Table S1 shows the proportion of missingness in each of the indicators for LCA.

Multivariable linear, logistic and Poisson regression models were computed to assess the association of each multimorbidity class with several outcomes at baseline and after 3 years of follow-up. Models for outcomes at baseline were adjusted for gender, age, years of schooling, household income and marital status (at baseline). Models for outcomes at follow-up were additionally adjusted for the same measure as the outcome at baseline. Regression models were conducted separately in one hundred imputed datasets and results combined using Rubin’s rules [36] in Stata SE version 13 (College Station, TX).

For sensitivity analysis, the same models described above were conducted again in the subsample of participants with valid values in all the variables (*n* = 1508) and compared with the results from the imputed datasets.

## Results

### Latent classes of multimorbidity pattern

Table 1 shows the adjusted BIC and CAIC values for the two to six-class models. There was an important drop in the adjusted BIC and CAIC values from the 2-class to the three-class model. The three-class model yielded the lowest CAIC value (CAIC = 1542.11) and, although the adjusted BIC corresponded to the four-class model (adjusted BIC = 1382.76), further inspection showed that one of the four classes presented a posterior probability lower than 0.7. Given the negligible difference between three and four-class models in terms of the adjusted BIC and following the parsimonious principle, the three-class model was finally chosen over the rest.

**Table 1:** Comparison between models

No. of latent classes	aBIC	CAIC
2	1542.06	1638.14
**3**	**1395.90**	**1542.11**
4	1382.76	1579.10
5	1389.11	1635.58
6	1384.05	1680.66

Note: Boldface type indicates the selected model. *aBIC* adjusted Bayesian Information Criterion, *CAIC* Consistent Akaike Information Criterion

Table 2 shows the distribution of sociodemographic, clinical and functioning characteristics in the overall sample at baseline and by multimorbidity classes. Some 63.8% of people were classified as being in the “healthy” class, with prevalence of all conditions below that observed in the whole sample. The “cardiorespiratory/mental/arthritis” class (6.2%) presented excess prevalence of depression (39.5%), arthritis (64.0%), asthma (73.2%), COPD (83.2%) and angina (40.0%). The “metabolic/stroke” class, which comprised 29.9% of the sample, showed excess prevalence of stroke (12.3%), obesity (55%), diabetes (35.1%) and hypertension (89.2%). The proportion of oral problems and cataract was high in both the “cardiovascular/mental/arthritis” and the “metabolic/stroke” classes.

**Table 2:** Baseline characteristics of the overall sample and people assigned to three multimorbidity classes

Characteristics	Total sample (*N* = 3541)	“Healthy” class (*n* = 2261, 63.8%)	“Cardiorespiratory/mental/arthritis” class (*n* = 220, 6.2%)	“Metabolic/stroke” class (*n* = 1060, 29.9%)	*P* value^a^
Age, *mean (SD)*	65.67 (10.28)	62.80 (9.46)	70.25 (9.86)	70.83 (9.67)	<0.001
Female, *n (%)*	1931 (54.5)	1166 (51.6)	126 (57.3)	639 (60.3)	<0.001
Years schooling, *mean (SD)*	9.94 (6.15)	11.19 (6.09)	6.96 (5.62)	7.88 (5.61)	<0.001
Widowed, *n (%)*	748 (21.1)	336 (14.9)	76 (34.5)	336 (31.7)	<0.001
Income (quintiles), *n (%)*					<0.001
1st (worst)	672 (21.1)	467 (23.2)	35 (17.0)	170 (17.5)	
2nd	668 (20.9)	323 (16.1)	73 (35.5)	272 (27.9)	
3rd	693 (21.7)	395 (19.6)	53 (25.7)	245 (25.2)	
4th	735 (23.0)	501 (24.9)	33 (16.0)	201 (20.6)	
5th (best)	424 (13.3)	326 (16.2)	12 (5.8)	86 (8.8)	
Comorbidities, *n (%)*					
Depression	415 (11.7)	113 (5.0)	87 (39.5)	215 (20.3)	<0.001
Arthritis	1051 (30.9)	319 (14.6)	130 (64.0)	602 (59.5)	<0.001
Asthma	287 (8.1)	91 (4.0)	161 (73.2)	35 (3.3)	<0.001
COPD	308 (8.7)	40 (1.8)	183 (83.2)	85 (8.0)	<0.001
Angina	275 (7.8)	46 (2.0)	88 (40.0)	141 (13.3)	<0.001
Stroke	147 (4.1)	7 (0.3)	10 (4.5)	130 (12.3)	<0.001
Diabetes	578 (16.3)	139 (6.1)	67 (30.6)	372 (35.1)	<0.001
Obesity	1089 (32.7)	448 (21.0)	94 (47.7)	547 (55.0)	<0.001
Edentulism	640 (18.1)	167 (7.4)	71 (32.3)	402 (37.9)	<0.001
Hypertension	2164 (63.2)	1096 (50.5)	142 (65.7)	926 (89.2)	<0.001
Cataract	828 (23.4)	196 (8.7)	104 (47.3)	528 (49.8)	<0.001
N° comorbidities, *mean (SD)*	2.20 (1.68)	1.18 (0.81)	5.17 (1.61)	3.76 (0.99)	<0.001
N° medical visits last 12 months, *mean (SD)*	4.05 (6.61)	3.04 (5.22)	7.02 (8.95)	5.55 (8.06)	<0.001
Hospital admission last 12 months, *n (%)*	790 (22.6)	374 (16.8)	98 (44.5)	318 (30.1)	<0.001
Limitations in ADLs, *n (%)*	343 (9.7)	79 (3.5)	68 (30.9)	196 (18.5)	<0.001
Limitations IADLs, *n (%)*	461 (13.0)	105 (4.6)	98 (44.5)	258 (24.3)	<0.001
WHODAS, *mean (SD)*	12.69 (18.07)	6.63 (11.78)	33.77 (24.26)	21.25 (20.74)	<0.001
WHOQOL-Age, *mean (SD)*	71.48 (14.86)	74.84 (13.04)	59.27 (17.40)	66.85 (15.37)	<0.001
ANIMAL TEST, *mean (SD)*	16.91 (7.62)	17.97 (7.89)	14.41 (6.74)	15.17(6.72)	<0.001
MEMORY score, *mean (SD)*	19.24 (7.07)	20.66 (7.00)	15.95 (6.32)	16.89 (6.50)	<0.001

*Note: COPD* Chronic Obstructive Pulmonary Disease, *ADL* Activities of Daily Living, *IADL* Instrumental Activities of Daily Living, *WHODAS* World Health Organization Disability Assessment Schedule 2.0, *WHOQOL-Age* World Health Organization Quality of Life instrument; Animal naming test scores ranged from 0 to 57; Memory scores ranged from 0 to 40

^a^Based on chi-squared tests for categorical variables and Kruskal-Wallis tests for continuous variables

The average posterior probabilities for all three classes exceeded 0.7 (0.85 for the “healthy” group, 0.81 for the “cardiorespiratory/mental/arthritis” class, and 0.75 for the “metabolic/stroke” group). Participants in the “healthy” class were more likely to be well-classified (79.7% of them had 0.7 or greater posterior probability). 68.6% and 60.9% of participants presented a posterior probability equal to or higher than 0.7 in the “cardiorespiratory/mental/arthritis” and the “metabolic/stroke” class, respectively.

People in the “metabolic/stroke” and “cardiorespiratory/mental/arthritis” latent groups were significantly older than those in the “healthy” group (Table 2). There were more females in the “metabolic/stroke” group, whereas the highest proportion of widows was seen in the “cardiorespiratory/mental/arthritis” class. This last group presented fewer years of schooling. The number of chronic conditions was significantly higher in the “cardiorespiratory/mental/arthritis” class. The prevalence of limitations in ADLs and IADLs was significantly higher in the “cardiorespiratory/mental/arthritis” class. The lowest level of quality of life was observed in the “cardiorespiratory/mental/arthritis” class, followed by the “metabolic/stroke” group. Level of disability was significantly higher in the “cardiorespiratory/mental/arthritis” and “metabolic/stroke” classes, compared with the healthy group. The lowest scores in verbal memory and verbal fluency at baseline were also seen in the “cardiorespiratory/mental/arthritis” class.

### Association between multimorbidity classes with outcomes at baseline and after 3 years

Table 3 displays the adjusted unstandardized coefficients for several outcomes assessed at baseline and follow-up. The “healthy” group was used as the reference group. At baseline, the two multimorbidity groups were associated with all the outcomes, except for verbal fluency. Being in the “cardiovascular/mental/arthritis” class was significantly related to lower levels of verbal memory, higher levels of disability, poorer quality of life, higher number of medical visits, higher risk of limitations in ADLs (OR = 7.91, 95%CI = 5.41–11.73, *p* < 0.001), IADLs (OR = 11.42, 95%CI = 8.04–16.22, *p* < 0.001) and more hospital admissions (OR = 3.77, 95%CI = 2.79–5.10, *p* < 0.001). Similarly, and compared with the healthy class, participants in the “metabolic/stroke” class were more likely to present poorer verbal memory and quality of life, higher levels of disability and number of medical visits in the last 12 months, greater odds for limitations in ADLs and IADLs, and being hospitalized more frequently. Inspection of ORs shows that the risk of presenting limitations in ADLs and IADLs and hospital admission was greater for people assigned to the “cardiovascular/mental/arthritis” group than for those assigned to the “metabolic/stroke” class.

**Table 3:** Association between latent multimorbidity membership and outcomes at baseline and follow-up

Outcomes	Associations at baseline^a^		Associations at follow-up^b^	
	Cardiorespiratory/mental/arthritis (*n* = 220) (b, SE)	Metabolic/stroke (*n* = 1060) (b, SE)	Cardiorespiratory/mental/arthritis (*n* = 220) (b, SE)	Metabolic/stroke^c^ (*n* = 1060) (b, SE)
Verbal fluency	−0.78 (0.50)	−0.17 (0.28)	−0.78 (0.55)	−0.78 (0.30)**
Verbal memory	−1.39 (0.44)**	−0.76 (0.25)**	−0.87 (0.53)	−0.12 (0.29)
Disability (WHODAS)	22.31 (1.10)***	10.08 (0.61)***	4.29 (1.38)	0.18 (0.75)
Quality of life	−13.59 (0.99)***	−6.85 (0.55)***	−5.49 (1.21)***	−1.75 (0.66)**
N° medical visits last 12 months	0.76 (0.10)***	0.54 (0.07)***	0.46 (0.15)**	0.22 (0.11)*
	Cardiorespiratory/mental/arthritis (OR, 95%CI)	Metabolic/stroke (OR, 95%CI)	Cardiorespiratory/mental/arthritis (OR, 95%CI)	Metabolic/stroke (OR, 95%CI)
Limitations in ADLs (yes/no)	7.97 (5.41–11.73)***	4.13 (3.07–5.54)***	4.05 (2.65–6.18)***	2.11 (1.64–2.72)***
Limitations in IADLs (yes/no)	11.42 (8.04–16.22)***	4.47 (3.45–5.80)***	2.17 (1.32–3.55)**	1.63 (1.17–2.29)**
Hospital admission last 12 months (yes/no)	3.77 (2.79–5.10)***	1.97 (1.63–2.38)***	1.88 (1.16–3.04)*	1.53 (1.10–2.12)*

*p < 0.05, **p < 0.01, ***p < 0.001

*Note:* The reference group for the multimorbidity group variable was the “healthy” class

Unstandardized coefficients from linear regression models for continuous outcomes (verbal fluency, memory, disability, quality of life), and from Poisson regression models for count data (n° medical visits)

Odds Ratios (95% confidence interval) from logistic regression models for binary outcomes (limitations in ADLs, IADLs and hospital admissions)

Models were run in 100 imputed datasets and results combined using Rubin’s rules

*ADLs* Activities of Daily Living, *IADLs* Instrumental Activities of Daily Living

^a^Adjusted for gender, age (at baseline), years of education (at baseline), marital status (at baseline) and income (at baseline);

^b^Adjusted for the same outcome measured at baseline, gender, age (at baseline), years of education (at baseline), marital status (at baseline) and income (at baseline)

In terms of cognitive function, verbal memory at follow-up was not significantly associated with multimorbidity groups. Only people in the “metabolic/stroke” class presented significantly lower levels of verbal fluency at follow-up, compared with the “healthy” group (b = −0.78, SE = 0.30; *p* < 0.01). Being in the “cardiorespiratory/mental/arthritis” or the “metabolic/stroke” groups was significantly associated with lower quality of life scores as well as a higher number of medical visits in the previous year. Persons classified in the “cardiorespiratory/mental/arthritis” group were four times more likely to have limitations in ADLs (OR = 4.05, 95%CI = 2.65–6.18) and twice as likely to present limitations in IADLs (OR = 2.17, 95%CI = 1.32–3.55) at follow-up. Membership of the “metabolic/stroke” class was significantly associated with greater risk of presenting limitations in ADLs (OR = 2.11, 95%CI = 1.64–2.72) and limitations in IADLs (OR = 1.63, 1.17–2.29) after 3 years. Both groups, compared with the “healthy” group, were at greater risk of hospital admission at follow-up.

### Sensitivity analysis

Regression models were run again using complete case analysis (*n* = 1508). In general, the results were similar to those obtained using multiple imputation. However, when using complete cases, the “cardiorespiratory/mental/arthritis” group was not associated with lower memory scores at baseline, and being in the “metabolic/stroke” class was not significantly related to lower verbal fluency scores at follow-up. Conversely, membership of the “cardiorespiratory/mental/arthritis” group appeared to be significantly related to disability at follow-up whereas this association was not significant when analyzing the imputed data (see Additional file 2: Table S2).

## Discussion

This study sought to describe multimorbidity patterns using LCA in a representative sample of Spanish community-dwelling adults over 50 years old. The LCA procedure identified three latent classes of multimorbidity which were statistically and clinically distinct, based on the presence or absence of eleven chronic conditions. These clusters generated using LCA were significantly related to several health, functioning and use of health service outcomes at baseline and were still significant predictors of most of them after 3 years of follow-up.

Previous studies conducting LCA to describe latent classes of co-occurring conditions in community samples of older people have yielded mixed results as regards the number of clusters detected. Four latent classes were identified in a cross-sectional sample of 4574 Australian seniors using eleven chronic conditions [6], including cancer, Parkinson’s disease, or osteoporosis. Another study found six clusters of multimorbidity in a sample of 14,502 people aged 65 years old and over, using 13 conditions, which also included neurological diseases, cancer and osteoporosis [11]. Comparison with these studies is difficult since the results might be influenced by the number and type of diseases included in the analysis, characteristics of the sample, or how data on diseases were collected. In our study, information about cancer, osteoporosis and dementia was not available. Conversely, we included other highly prevalent conditions such as edentulism, cataract and obesity.

The majority of our sample (63.8%) was classified into the “healthy” class. This latent group has previously been reported in other studies which also conducted LCA [6, 11]. However, the proportion classified as “healthy” in our study is larger than that described in these studies. This difference could be explained by the age of participants. For example, Whitson et al. [11] found that 32.8% of their community sample was classified in the minimal disease category but the average age was older than in our study (76.4 vs. 65.7 years). Our findings support the existence of broad multimorbidity patterns. These clusters are very similar to those reported in a review where 14 studies on patterns of multimorbidity were considered [12]. Despite the fact that there was considerable heterogeneity between studies in terms of number and types of chronic conditions included or the statistical approach used, the authors concluded that there are at least three broad patterns; one comprising cardiovascular and metabolic diseases, a second one related to mental health problems and a third including musculoskeletal disorders. In our study, 30% of participants were classified under the “metabolic/stroke” category. This cluster is close to the “metabolic syndrome” which has been shown to increase the risk of stroke and diabetes [37]. The least frequent latent group was the “cardiorespiratory/mental/arthritis” class. However, it appeared to be the most severe category, with worst functioning and greatest burden. This category clusters a large number of pathologies together (i.e., angina, COPD, asthma, depression and arthritis). Previous studies also reported a cluster of angina and respiratory diseases [17]. The co-occurrence of health problems and musculoskeletal disorders has been consistently reported [38, 39] and has been associated with a constellation of comorbidities. However, the link between mental health conditions and COPD is still unclear [40].

Cataract and edentulism were both highly prevalent in the two multimorbidity clusters. In a similar study, visual impairment was associated with a history of stroke, diabetes and arthritis [4, 41]. Patients with arthritis are more likely to suffer from cataract, after adjusting for glucocorticoid intake [42]. Heart diseases have also been related to higher risk for cataract [43], supporting the theory of inflammatory pathways [44]. The presence of edentulism has been related to diabetes, coronary artery disease, hypertension, and rheumatoid arthritis [45].

Several underlying mechanisms could explain the nonrandom association between chronic conditions. Insulin resistance has been proposed as one possible underlying mechanism explaining the strong association between metabolic syndrome and stroke, affecting metabolic processes and leading to abnormalities of vascular reactivity [37]. A change of lifestyles to reduce metabolic syndrome can help prevent stroke and other vascular diseases. Other therapeutic strategies can include targeting insulin resistance [37]. The association between respiratory diseases and coronary heart disease has previously been established [46] and could be explained by inflammation, hypoxia, or stress processes. Other environmental risk factors could include smoking or air pollution [17]. Unexpected associations between conditions (such as arthritis and respiratory diseases) should be studied in the future. It has been suggested that medication could be a risk factor for co-occurrence of certain groups of diseases [12]. Future research should focus on finding the underlying pathogenesis connecting these medical conditions, and the shared risk factors.

Our findings show that these three multimorbidity patterns are qualitatively distinct with important differences with respect to sociodemographic, clinical and functioning aspects. Being older, female and having a low educational level have been consistently associated with more risk of suffering from multimorbidity [47, 48]. In common with other studies [4], our results show that multimorbidity clusters were cross-sectionally associated with a greater degree of disability, poor functioning, lower quality-of-life levels, poor memory function and greater risk of health care visits and hospitalizations. Persons assigned to one of these two multimorbidity clusters still presented poor quality of life, were more likely to present problems in independent living, and used health services more frequently (including being hospitalized) after 3 years of follow-up.

One interesting result is the significant association between multimorbidity class membership and cognitive function. Worse verbal fluency at follow-up was only linked to membership of the “metabolic/stroke” class. Depression, which is part of the “cardiovascular/mental/arthritis” cluster, has been consistently linked to memory complaints [49] as well as worse performance in memory tests [50]. Previous longitudinal studies have also reported an association between musculoskeletal diseases, lung diseases or arthritis, with cognitive decline [51]. Vascular risk factors and vascular diseases, such as stroke, have been consistently associated with cognitive deterioration in older adults [52]. Similarly, Ganguli et al. [53] found cross-sectional associations between a history of stroke, diabetes and abdominal adiposity with worse memory and executive function in a population-based cohort of old people, although these associations were not observed after 4 years of follow-up. The authors suggested that the effect of these diseases on brain structures would be static rather than progressive [53]. Performance in memory tests might also be affected by the learning effect [54]. Longer follow-up periods are needed to observe possible cognitive decline over time associated with these multimorbidity clusters and to avoid possible learning effects.

Some limitations should be considered when interpreting our findings. In our study, some diseases were not evaluated (such as cancer or neurological diseases) and the inclusion of additional chronic conditions might have yielded some different patterns. The COURAGE protocol included a limited number of chronic conditions based on their high prevalence and impact on health outcomes. Conversely, depression and obesity were considered in our study, although they are commonly omitted in other research. Second, the presence of chronic diseases was partially based on self-reporting, and can thus be affected by measurement errors or lack of accuracy. However, the literature shows that self-reported measures of chronic diseases are widely used in large population-based studies and show reasonable accuracy [55–57]. Moreover, we used additional questions about symptoms during the interview, allowing us to detect undiagnosed cases. For hypertension and obesity, objective measures were obtained during the interview. Next, misclassification of persons assigned to each latent class is reasonable [31]. However, there is still some degree of uncertainty associated with latent class membership and results should be interpreted with caution [27]. Finally, the use of multiple imputations could add some bias. Nevertheless, the sensitivity analysis showed similar results. The few differences between results with complete cases and imputed data might be explained by greater change variation when using the former, and because under MAR assumption, multiple imputation should correct biases that may arise in complete cases analyses [58].

## Conclusions

This study identified three qualitatively separate, broad multimorbidity clusters using LCA in a Spanish nationally representative sample of older adults with distinct clinical and sociodemographic characteristics. The latent classes identified presented relatively low misclassification errors, and demonstrated predictive and external validity. Multimorbidity has been consistently related to greater burden and increased use of health services. The single-disease paradigm does not seem to fit the majority of persons with more than one chronic condition. Future efforts should focus on the underlying mechanisms of these multimorbidity clusters (lifestyles, metabolic or inflammatory factors, stress, and environmental factors) and determine targets for prevention and intervention.

## Additional files

Additional file 1: Table S1. Proportion of missingness for indicators of latent classes and variables included in the multiple imputation model [59–61]. (DOC 76 kb)

Additional file 2: Table S2. Association between latent multimorbidity membership with outcomes at baseline and follow-up in the completers (*n* = 1508). (DOCX 17 kb)

## Abbreviations

95%CI: 95% confidence interval
ADLs: Activities of daily living
BIC: Bayesian Information Criterion
BMI: Body Mass Index
CAIC: Corrected Akaike Information Criterion
CIDI: Composite International Diagnostic Interview
COPD: Chronic obstructive pulmonary disease
COURAGE: Collaborative Research on Ageing in Europe
DSM-IV: Diagnostic and Statistical Manual of Mental Disorders
FIML: Full-information maximum likelihood
IADLs: Instrumental activities of daily living
ICE: Imputation by chained equations
LCA: Latent class analysis
MAR: Missing-at-random
OR: Odds Ratio
SE: Standard error
WHODAS 2.0: World Health Organization Disability Assessment Schedule 2.0
WHOQOL: World Health Organization Quality of Life instrument
