# Supplementary material for: Latent class analysis of multimorbidity patterns and associated outcomes in Spanish older adults: a prospective cohort study
Source: BMC Geriatr. 2017 Aug 18;17:186. doi: 10.1186/s12877-017-0586-1 (PMC5563011; doi:10.1186/s12877-017-0586-1)
Supplement: Supplementary file 1 — Proportion of missingness for indicators of latent classes and variables included in the multiple imputation model [59–61]. (DOC 76 kb) [file 12877_2017_586_MOESM1_ESM.doc]

**Table S1. Proportion of missingness for indicators of latent classes^a^ and variables included in the multiple imputation model^b^**

| 1. Indicators of multimorbidity classes | | |
| --- | --- | --- |
|  | n (%) missingness  Baseline | n (%) missingness  Follow-up |
| Depression | 0 | na |
| Arthritis | 144 (4.1%) | na |
| Asthma | 0 | na |
| COPD | 0 | na |
| Angina | 0 | na |
| Stroke | 0 | na |
| Diabetes | 1 (0.03%) | na |
| Obesity | 213 (6.0%) | na |
| Edentulism | 0 | na |
| Hypertension | 118 (3.3%) | na |
| Cataract | 0 | na |
| 1. Variables used in the regression models | | |
| Gender | 0 | na |
| Age | 0 | na |
| Years of schooling | 26 (0.7%) | na |
| Marital status | 0 | na |
| Memory function (0-40) | 169 (4.8%) | 1754 (49.5%) |
| Verbal fluency (0-57) | 0 | 1717 (48.5%) |
| WHODAS (0-100) | 0 | 1657 (46.8%) |
| Quality of life (0-100) | 0 | 1655 (46.7%) |
| Limitations in ADLs (yes/no) | 0 | 1655 (46.7%) |
| Limitations in IADLs (yes/no) | 0 | 1655 (46.7%) |
| Nº hospital admissions last 12 months | 39 (1.1%) | 1869 (52.8%) |
| Nº hospitalizations last 12 months | 39 (1.1%) | 1884 (53.2%) |
| Latent multimorbidity classes | 0 | na |
| Income (quintiles) | 349 (9.8%) | na |
| 1. Auxiliary variables | | |
| Depression (yes/no) | 0 | na |
| Physical activity | 0 | na |
| Grip strength (yes/no) | 463 (13.1%) | na |
| Health (0-100) | 0 | na |
| Mobility (0-100) | 257 (7.2%) | na |
| Currently smoking (yes/no) | 0 | na |
| Heavy drinker (yes/no) | 0 | na |

na= not applicable

Income=quintiles of household income, first indicating lowest wealth levels.

Physical activity=(0) high, (1) moderate, (2) low, based on the Global Physical Activity Questionnaire [59].

Grip strength= (0) normal, (1) weak, measured with a dynamometer. Cut-off points were based on Cruz-Jentoft et al. [60].

Health= one single factor score calculated by Rash models [61]. Higher scores indicate better health.

Mobility= based on a single factor score of mobility items from the COURAGE protocol (Leonardi et al., 2004). Higher scores indicate better mobility.
