# Supplementary material for: Latent class analysis of multimorbidity patterns and associated outcomes in Spanish older adults: a prospective cohort study
Source: BMC Geriatr. 2017 Aug 18;17:186. doi: 10.1186/s12877-017-0586-1 (PMC5563011; doi:10.1186/s12877-017-0586-1)
Supplement: Supplementary file 2 — Association between latent multimorbidity membership with outcomes at baseline and follow-up in the completers (n = 1508). (DOCX 17 kb) [file 12877_2017_586_MOESM2_ESM.docx]

**Table S2.** Association between latent multimorbidity membership with outcomes at baseline and follow-up in the completers (*n*=1,508)

|  | Associations at baseline^a^ | | |  | Associations at follow-up^b^ | | |
| --- | --- | --- | --- | --- | --- | --- | --- |
| Outcomes | Cardiorespiratory/  mental/arthritis  (b, SE) | Metabolic/stroke  (b, SE) | R^2^ |  | Cardiorespiratory/  mental/arthritis  (b, SE) | Metabolic/stroke  (b, SE) | R^2^ |
| Verbal fluency | -1.02 (0.69) | -0.70 (0.40) | 0.23 |  | -0.59 (0.57) | -0.60 (0.33) | 0.42 |
| Verbal memory | -1.08 (0.64) | -0.84 (0.37)* | 0.27 |  | -0.55 (0.55) | -0.04 (0.31) | 0.49 |
| Disability (WHODAS) | 19.88 (1.56)*** | 9.62 (0.89)*** | 0.30 |  | 4.86 (1.53)** | 0.46 (0.87) | 0.46 |
| Quality of life | -14.28 (1.45)*** | -6.85 (0.83)*** | 0.18 |  | -5.23 (1.33)*** | -1.73 (0.77)* | 0.34 |
| Nº medical visits last 12 months | 0.48 (0.05)*** | 0.34 (0.03)*** | 0.04 |  | 0.43 (0.05)*** | 0.17 (0.03)*** | 0.05 |
|  | Cardiorespiratory/  mental/arthritis  (OR, 95%CI) | Metabolic/stroke  (OR, 95%CI) | Pseudo R^2^ |  | Metabolic/stroke  (OR, 95%CI) | Metabolic/stroke  (OR, 95%CI) | Pseudo R^2^ |
| Limitations in ADLs | 10.81 (5.62-20.79)*** | 6.41 (3.83-10.90)*** | 0.19 |  | 4.90 (2.94-8.18)*** | 1.81 (1.33-2.45)*** | 0.22 |
| Limitations in IADLs | 13.22 (7.59-23.03)*** | 4.92 (3.19-7.59)*** | 0.17 |  | 2.13 (1.16-3.92)* | 1.54 (1.01-2.34)* | 0.26 |
| Hospital admission last 12 months | 2.56 (1.61-4.08)*** | 1.594 (1.18-2.13)** | 0.02 |  | 2.45 (1.39-4.33)** | 1.62 (1.12-2.35)* | 0.04 |

**p*<0.05, ***p*<0.01, ****p*<0.001

*Note:* The reference group for the multimorbidity group variable was the “healthy” class.

Unstandardized coefficients from linear regression models for continuous outcomes (verbal fluency, memory, disability, quality of life) and from Poisson regression models for count data (nº medical visits).

Odds Ratios (95% confidence interval) from logistic regression models for binary outcomes (limitations in ADLs, IADLs and hospital admissions).

ADLs= Activities of Daily Living, IADLs= Instrumental Activities of Daily Living

^a^Adjusted for gender, age (at baseline), years of education (at baseline), marital status (at baseline) and income (at baseline).

^b^Adjusted for the same outcome measured at baseline, gender, age (at baseline), years of education (at baseline), marital status (at baseline) and income (at baseline).
